# {4,4′-Dibromo-2,2′-[2,2-dimethyl­propane-1,3-diylbis(nitrilo­methanylyl­idene)]diphenolato-κ^4^ *O*,*N*,*N*′,*O*′}nickel(II)

**DOI:** 10.1107/S1600536811009056

**Published:** 2011-03-15

**Authors:** Saeed Rayati, Akbar Ghaemi, Behrouz Notash

**Affiliations:** a Department of Chemistry, K. N. Toosi University of Technology, PO Box 16315-1618, Tehran, Iran; b Department of Chemistry, Islamic Azad University, Saveh Branch, PO Box 39187-366, Saveh, Iran; c Department of Chemistry, Shahid Beheshti University, G. C. Evin, Tehran 1983963113, Iran

## Abstract

In the title compound, [Ni(C_19_H_18_Br_2_N_2_O_2_)], the Ni^II^ ion, lying on a twofold rotation axis, is coordinated by two N atoms and two O atoms from the Schiff base ligand in a distorted square-planar geometry. Weak inter­molecular C—H⋯O hydrogen bonds stabilize the crystal structure.

## Related literature

For the catalytic properties of Schiff base complexes, see: Cozzi (2004 ▶). For related structures see: Fun *et al.* (2008 ▶); Kargar *et al.* (2008 ▶). For the synthesis of the ligand, see: Fairhurst *et al.* (1995 ▶).
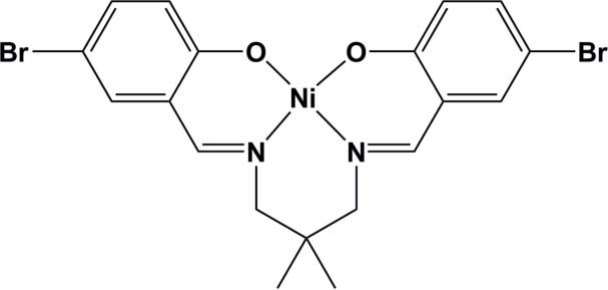


## Experimental

### 

#### Crystal data

[Ni(C_19_H_18_Br_2_N_2_O_2_)]
*M* *_r_* = 524.84
Monoclinic, 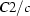

*a* = 24.227 (6) Å
*b* = 11.030 (3) Å
*c* = 7.535 (2) Å
β = 107.939 (19)°
*V* = 1915.6 (9) Å^3^
*Z* = 4
Mo *K*α radiation
μ = 5.20 mm^−1^
*T* = 298 K
0.30 × 0.20 × 0.15 mm

#### Data collection

Stoe IPDS-2 diffractometer
Absorption correction: numerical (*X-SHAPE* and *X-RED32*; Stoe & Cie, 2005 ▶) *T*_min_ = 0.289, *T*_max_ = 0.449
7514 measured reflections
2575 independent reflections
1892 reflections with *I* > 2σ(*I*)
*R*_int_ = 0.142

#### Refinement

*R*[*F* ^2^ > 2σ(*F* ^2^)] = 0.073
*wR*(*F* ^2^) = 0.211
*S* = 1.16
2575 reflections
119 parameters
H-atom parameters constrained
Δρ_max_ = 0.74 e Å^−3^
Δρ_min_ = −0.99 e Å^−3^

### 

Data collection: *X-AREA* (Stoe & Cie, 2005 ▶); cell refinement: *X-AREA*; data reduction: *X-AREA*; program(s) used to solve structure: *SHELXS97* (Sheldrick, 2008 ▶); program(s) used to refine structure: *SHELXL97* (Sheldrick, 2008 ▶); molecular graphics: *ORTEP-3* (Farrugia, 1997 ▶); software used to prepare material for publication: *WinGX* (Farrugia, 1999 ▶).

## Supplementary Material

Crystal structure: contains datablocks I, global. DOI: 10.1107/S1600536811009056/hy2415sup1.cif

Structure factors: contains datablocks I. DOI: 10.1107/S1600536811009056/hy2415Isup2.hkl

Additional supplementary materials:  crystallographic information; 3D view; checkCIF report

## Figures and Tables

**Table 1:** Selected bond lengths (Å)

Ni1—N1	1.874 (4)
Ni1—O1	1.856 (4)

**Table 2:** Hydrogen-bond geometry (Å, °)

*D*—H⋯*A*	*D*—H	H⋯*A*	*D*⋯*A*	*D*—H⋯*A*
C3—H3*B*⋯O1^i^	0.97	2.40	3.210 (6)	141

Symmetry code: (i) 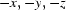.

**Fig. 1.**
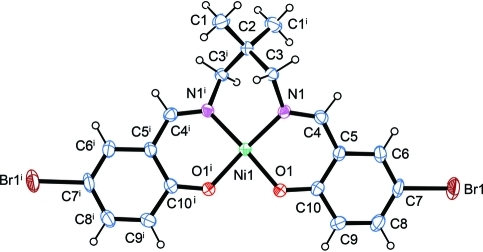
Molecular structure of the title compound, with displacement ellipsoids drawn at the 30% probability level. [Symmetry code: (i) -x, y, -z+1/2].

**Fig. 2.**
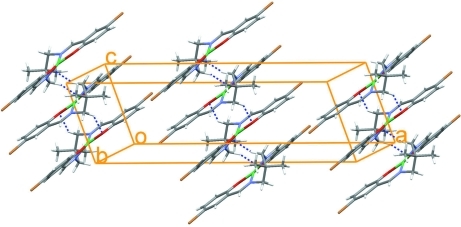
The packing diagram of the title compound. Hydrogen bonds are shown as blue dashed lines.

## supplementary crystallographic information

### Comment

Schiff base complexes are found to exhibit large applications such as catalytic properties (Cozzi, 2004). *N*,*N*'-Bis(5-bromo-2-hydroxybenzylidene)- 2,2-dimethylpropane-1,3-diamine ligand has been previously synthesized and structurally characterized by X-ray diffraction (Fun *et al.*, 2008). The structure of a copper(II) complex of this Schiff base ligand has been also reported by Fun's group (Kargar *et al.*, 2008).

Herein, we report the synthesis and crystal structure of an Ni(II) complex with this Schiff base ligand. The molecular structure of the title compound is shown in Fig. 1. The asymmetric unit of the title compound contains half of the molecule. The Ni^II^ ion, lying on a twofold rotation axis, is coordinated by two N atoms and two O atoms from a Schiff base ligand (Table 1). The coordination environment around the Ni^II^ ion can be described as distorted squar-planar. In the crystal, weak intermolecular C—H···O hydrogen bonds stabilize the structure (Table 2, Fig. 2).

### Experimental

*N*,*N*'-Bis(5-bromo-2-hydroxybenzylidene)- 2,2-dimethylpropane-1,3-diamine was prepared according to the described procedure (Fairhurst *et al.*, 1995). To a stirred ethanolic solution (30 ml) of 2,2-dimethylpropylenediamine (0.102 g, 1 mmol), 5-bromo-2-hydroxybenzaldehyde (0.402 g, 2 mmol) was added. The bright yellow solution was stirred and heated to reflux for 1 h. A yellow precipitate was obtained that was filtered off, washed with diethyl ether (yield: 70%; m.p.: 140 °C).

The title complex was prepared by the following procedure. The Schiff base ligand (0.467 g, 1 mmol) was dissolved in 20 ml ethanol. A solution of nickel(II) acetate (0.248 g, 1 mmol) in ethanol was added to the solution of ligand and the reaction mixture was refluxed for 1 h. The colored solution was concentrated to yield brown powders. The product washed with ethanol and air dried (yield: 95%; decomposition temperature: 242°C).

### Refinement

H atoms were positioned geometrically and refined as riding atoms, with C—H = 0.93 (aromatic), 0.97 (CH_2_) and 0.96 (CH_3_) Å and with *U*_iso_(H) = 1.2 or 1.5*U*_eq_(C).

### Crystal data

**Table:**

[Ni(C_19_H_18_Br_2_N_2_O_2_)]	*F*(000) = 1040.0
*M**_r_* = 524.84	*D*_x_ = 1.820 Mg m^−^^3^
Monoclinic, *C*2/*c*	Mo *K*α radiation, λ = 0.71073 Å
Hall symbol: -C 2yc	Cell parameters from 2575 reflections
*a* = 24.227 (6) Å	θ = 3.2–29.2°
*b* = 11.030 (3) Å	µ = 5.20 mm^−^^1^
*c* = 7.535 (2) Å	*T* = 298 K
β = 107.939 (19)°	Plate, brown
*V* = 1915.6 (9) Å^3^	0.30 × 0.20 × 0.15 mm
*Z* = 4	

### Data collection

**Table:**

Stoe IPDS-2 diffractometer	2575 independent reflections
Radiation source: fine-focus sealed tube	1892 reflections with *I* > 2σ(*I*)
graphite	*R*_int_ = 0.142
ω scans	θ_max_ = 29.2°, θ_min_ = 3.2°
Absorption correction: numerical (*X-SHAPE* and *X-RED32*; Stoe & Cie, 2005)	*h* = −33→24
*T*_min_ = 0.289, *T*_max_ = 0.449	*k* = −15→13
7514 measured reflections	*l* = −10→10

### Refinement

**Table:**

Refinement on *F*^2^	Primary atom site location: structure-invariant direct methods
Least-squares matrix: full	Secondary atom site location: difference Fourier map
*R*[*F*^2^ > 2σ(*F*^2^)] = 0.073	Hydrogen site location: inferred from neighbouring sites
*wR*(*F*^2^) = 0.211	H-atom parameters constrained
*S* = 1.16	*w* = 1/[σ^2^(*F*_o_^2^) + (0.0955*P*)^2^ + 1.9321*P*] where *P* = (*F*_o_^2^ + 2*F*_c_^2^)/3
2575 reflections	(Δ/σ)_max_ < 0.001
119 parameters	Δρ_max_ = 0.74 e Å^−^^3^
0 restraints	Δρ_min_ = −0.98 e Å^−^^3^

### Fractional atomic coordinates and isotropic or equivalent isotropic displacement parameters (Å^2^)

**Table:**

	*x*	*y*	*z*	*U*_iso_*/*U*_eq_	
C1	0.0516 (4)	0.4164 (6)	0.2433 (9)	0.0606 (17)	
H1A	0.0407	0.4647	0.1319	0.073*	
H1B	0.0836	0.3650	0.2432	0.073*	
H1C	0.0629	0.4685	0.3505	0.073*	
C2	0.0000	0.3381 (7)	0.2500	0.0386 (14)	
C3	−0.0173 (2)	0.2567 (5)	0.0764 (6)	0.0376 (10)	
H3A	−0.0428	0.3023	−0.0268	0.045*	
H3B	0.0173	0.2364	0.0438	0.045*	
C4	−0.0989 (2)	0.1277 (5)	−0.0094 (7)	0.0411 (11)	
H4	−0.1172	0.1946	−0.0780	0.049*	
C5	−0.1317 (2)	0.0172 (5)	−0.0337 (7)	0.0425 (11)	
C6	−0.1902 (3)	0.0171 (7)	−0.1506 (8)	0.0546 (14)	
H6	−0.2078	0.0894	−0.2018	0.065*	
C7	−0.2206 (3)	−0.0880 (7)	−0.1878 (9)	0.0591 (17)	
C8	−0.1955 (3)	−0.1976 (7)	−0.1126 (9)	0.0587 (16)	
H8	−0.2172	−0.2687	−0.1381	0.070*	
C9	−0.1388 (3)	−0.2006 (6)	−0.0009 (8)	0.0518 (14)	
H9	−0.1223	−0.2746	0.0462	0.062*	
C10	−0.1048 (2)	−0.0934 (5)	0.0447 (7)	0.0404 (11)	
N1	−0.04685 (19)	0.1431 (4)	0.0982 (5)	0.0357 (9)	
O1	−0.05103 (17)	−0.1011 (3)	0.1491 (5)	0.0427 (8)	
Ni1	0.0000	0.02440 (9)	0.2500	0.0336 (3)	
Br1	−0.29838 (4)	−0.08575 (11)	−0.35030 (14)	0.0979 (5)	

### Atomic displacement parameters (Å^2^)

**Table:**

	*U*^11^	*U*^22^	*U*^33^	*U*^12^	*U*^13^	*U*^23^
C1	0.074 (5)	0.048 (4)	0.053 (3)	−0.013 (3)	0.010 (3)	0.002 (3)
C2	0.037 (3)	0.038 (4)	0.036 (3)	0.000	0.004 (3)	0.000
C3	0.041 (3)	0.035 (3)	0.036 (2)	0.000 (2)	0.0104 (19)	0.0032 (18)
C4	0.046 (3)	0.040 (3)	0.036 (2)	0.004 (2)	0.0100 (19)	0.0043 (19)
C5	0.038 (2)	0.046 (3)	0.039 (2)	−0.005 (2)	0.0036 (19)	−0.001 (2)
C6	0.040 (3)	0.058 (4)	0.054 (3)	0.000 (3)	−0.003 (2)	0.008 (3)
C7	0.031 (3)	0.073 (5)	0.060 (3)	−0.014 (3)	−0.007 (2)	−0.003 (3)
C8	0.045 (3)	0.060 (4)	0.064 (3)	−0.018 (3)	0.007 (3)	−0.005 (3)
C9	0.057 (4)	0.039 (3)	0.055 (3)	−0.007 (3)	0.011 (3)	−0.003 (2)
C10	0.040 (3)	0.044 (3)	0.036 (2)	−0.005 (2)	0.0105 (19)	−0.0033 (19)
N1	0.040 (2)	0.036 (2)	0.0277 (15)	−0.0035 (18)	0.0057 (14)	−0.0005 (14)
O1	0.0371 (19)	0.0349 (19)	0.0495 (19)	0.0004 (15)	0.0035 (15)	−0.0030 (14)
Ni1	0.0339 (5)	0.0315 (5)	0.0325 (4)	0.000	0.0060 (3)	0.000
Br1	0.0519 (5)	0.1002 (8)	0.1070 (7)	−0.0251 (5)	−0.0265 (4)	0.0188 (5)

### Geometric parameters (Å, °)

**Table:**

C1—C2	1.533 (8)	C5—C6	1.421 (8)
C1—H1A	0.9600	C5—C10	1.423 (8)
C1—H1B	0.9600	C6—C7	1.354 (10)
C1—H1C	0.9600	C6—H6	0.9300
C2—C1^i^	1.533 (8)	C7—C8	1.393 (11)
C2—C3	1.535 (7)	C7—Br1	1.906 (6)
C2—C3^i^	1.535 (7)	C8—C9	1.372 (9)
C3—N1	1.476 (7)	C8—H8	0.9300
C3—H3A	0.9700	C9—C10	1.423 (8)
C3—H3B	0.9700	C9—H9	0.9300
C4—N1	1.284 (7)	C10—O1	1.300 (7)
C4—C5	1.435 (8)	Ni1—N1	1.874 (4)
C4—H4	0.9300	Ni1—O1	1.856 (4)
			
C2—C1—H1A	109.5	C7—C6—H6	119.9
C2—C1—H1B	109.5	C5—C6—H6	119.9
H1A—C1—H1B	109.5	C6—C7—C8	121.2 (6)
C2—C1—H1C	109.5	C6—C7—Br1	119.2 (5)
H1A—C1—H1C	109.5	C8—C7—Br1	119.5 (5)
H1B—C1—H1C	109.5	C9—C8—C7	119.9 (6)
C1—C2—C1^i^	111.4 (8)	C9—C8—H8	120.0
C1—C2—C3	108.3 (3)	C7—C8—H8	120.0
C1^i^—C2—C3	110.2 (3)	C8—C9—C10	121.7 (6)
C1—C2—C3^i^	110.2 (3)	C8—C9—H9	119.2
C1^i^—C2—C3^i^	108.3 (3)	C10—C9—H9	119.2
C3—C2—C3^i^	108.4 (6)	O1—C10—C5	123.6 (5)
N1—C3—C2	114.6 (4)	O1—C10—C9	119.4 (5)
N1—C3—H3A	108.6	C5—C10—C9	117.0 (5)
C2—C3—H3A	108.6	C4—N1—C3	117.2 (4)
N1—C3—H3B	108.6	C4—N1—Ni1	125.9 (4)
C2—C3—H3B	108.6	C3—N1—Ni1	116.0 (3)
H3A—C3—H3B	107.6	C10—O1—Ni1	128.0 (4)
N1—C4—C5	126.2 (5)	O1—Ni1—O1^i^	83.5 (2)
N1—C4—H4	116.9	O1—Ni1—N1^i^	166.70 (17)
C5—C4—H4	116.9	O1^i^—Ni1—N1^i^	93.97 (18)
C6—C5—C10	119.9 (6)	O1—Ni1—N1	93.97 (18)
C6—C5—C4	119.1 (6)	O1^i^—Ni1—N1	166.70 (17)
C10—C5—C4	120.7 (5)	N1^i^—Ni1—N1	91.3 (3)
C7—C6—C5	120.2 (6)		
			
C1—C2—C3—N1	154.4 (5)	C8—C9—C10—C5	−1.2 (9)
C1^i^—C2—C3—N1	−83.4 (6)	C5—C4—N1—C3	168.8 (5)
C3^i^—C2—C3—N1	34.9 (3)	C5—C4—N1—Ni1	0.5 (7)
N1—C4—C5—C6	176.4 (5)	C2—C3—N1—C4	118.2 (5)
N1—C4—C5—C10	−8.9 (8)	C2—C3—N1—Ni1	−72.3 (5)
C10—C5—C6—C7	0.0 (9)	C5—C10—O1—Ni1	9.6 (7)
C4—C5—C6—C7	174.6 (6)	C9—C10—O1—Ni1	−172.6 (4)
C5—C6—C7—C8	0.3 (11)	C10—O1—Ni1—O1^i^	179.3 (5)
C5—C6—C7—Br1	−178.4 (5)	C10—O1—Ni1—N1^i^	99.5 (8)
C6—C7—C8—C9	−1.0 (11)	C10—O1—Ni1—N1	−13.9 (4)
Br1—C7—C8—C9	177.7 (5)	C4—N1—Ni1—O1	8.7 (4)
C7—C8—C9—C10	1.5 (10)	C3—N1—Ni1—O1	−159.7 (3)
C6—C5—C10—O1	178.3 (5)	C4—N1—Ni1—O1^i^	87.3 (9)
C4—C5—C10—O1	3.7 (8)	C3—N1—Ni1—O1^i^	−81.1 (9)
C6—C5—C10—C9	0.4 (8)	C4—N1—Ni1—N1^i^	−159.1 (5)
C4—C5—C10—C9	−174.1 (5)	C3—N1—Ni1—N1^i^	32.5 (3)
C8—C9—C10—O1	−179.1 (6)		

Symmetry codes: (i) −*x*, *y*, −*z*+1/2.

### Hydrogen-bond geometry (Å, °)

**Table:**

*D*—H···*A*	*D*—H	H···*A*	*D*···*A*	*D*—H···*A*
C3—H3B···O1^ii^	0.97	2.40	3.210 (6)	141

Symmetry codes: (ii) −*x*, −*y*, −*z*.
